# Jagged-2 enhances immunomodulatory activity in adipose derived mesenchymal stem cells

**DOI:** 10.1038/srep14284

**Published:** 2015-09-28

**Authors:** Zhu Xishan, Zhang Bin, Zhao Haiyue, Dou Xiaowei, Bai Jingwen, Zhang Guojun

**Affiliations:** 1 The Breast Center, Cancer Hospital, Shantou University Medical College, Shantou, China; 2 Institute of Basic medicine. Peking Union Medical College, Chinese Academy of Medical Science, China; 3 Clinical department, Capital Medical University

## Abstract

Adipose derived Mesenchymal stem cells (AMSCs) are able to expand *in vitro* and undergo differentiation into multiple cell lineages, yet have low immunogenicity while exhibiting several immunoregulatory characteristics. We sought to investigate the immunomodulatory mechanisms of AMSCs to better understand their immunogenic properties. Following 10 days of chondrogenic differentiation or 48 hours of IFN-γ pretreatment, AMSCs retained low level immunogenicity but prominent immunoregulatory activity and AMSC immunogenicity was enhanced by chondrogenic differentiation or IFN-γ treatment. We found Jagged-2 expression was significantly elevated following chondrogenic differentiation or IFN-γ pretreatment. Jagged-2-RNA interference experiments suggested that Jagged-2-siRNA2 suppresses Jagged-2 expression during chondrogenic differentiation and in IFN-γ pretreated AMSCs. Besides, Jagged-2 interference attenuated immunosuppressive activity by mixed lymphocyte culture and mitogen stimulation experiments. So, the immunoregulatory activity of AMSCs, to some extent dependent upon Jagged-2, might be stronger after multilineage differentiation or influence from inflammatory factors. This may also be why rejection does not occur after allogeneic AMSCs differentiate into committed cells.

Mesenchymal stem cells (MSCs) are multipotential nonhematopoietic progenitor cells isolated from many adult tissues, in particular the bone marrow and adipose tissue^1^,^2^,^3^. Along with their capacity for differentiating into cells of mesodermal lineage such as adipocytes, osteoblasts, and chondrocytes, they have also generated great interest for their ability to display immunomodulatory functions^4^,^5^. Indeed, a major breakthrough came with the finding that they are able to induce peripheral tolerance, suggesting they may be used as therapeutic tools in immune-mediated disorders^6^.

MSCs are adult stem cells traditionally found in the bone marrow. However, MSCs can also be isolated from other tissues including adipose, cord blood, peripheral blood, fallopian tube, and fetal liver and lung^7^,^8^,^9^. In addition to their *in vitro* expansion and multilineage differentiation, MSCs have low immunogenicity and immunoregulatory properties^10^. Thus, there are promising preventative or therapeutic applications of MSCs for the repair of tissue damage, treatment of autoimmune disease, and induction of allogeneic transplantation tolerance^11^,^12^,^13^. Because of their low-level expression of MHC-I (major histocompatibility complex-1) antigens, lack of MHC-II and costimulatory molecules CD80, CD86, and CD40 expression, MSCs escape immune surveillance and can repair allogeneic tissue damage^14^. Although MSCs do not express MHC antigens and costimulatory molecules *in vitro*, they are expressed after they differentiate into committed cells during allogeneic tissue damage repair *in vivo*. Why such cells are not rejected by the host cannot be explained by their lack of immunogenicity. However, the mechanisms governing these phenomena are poorly understood.

Based on these observations, we sought to investigate adipose derived MSCs (AMSCs) for immunogenic properties and immunomodulatory mechanisms to provide new data for their clinical application. We first used an *in vitro* chondrogenic differentiation or gamma interferon (IFN)-γ pretreatment model to simulate the differentiation microenvironment or inflammation *in vivo* and explore changes in AMSC immunological properties and potential IFN-γ regulated mechanisms in osteogenesis. In addition, we also examined why rejection does not occur after allogeneic MSCs differentiate into committed host cells.

## Results

### The biological characteristics of AMSCs

**Figure 1:**
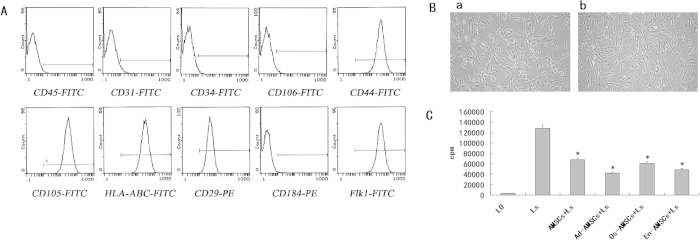
Biological characteristics of AMSCs. (**A**) The morphology of AMSCs (a: before Jagged-2 RNAi; b:after Jagged-2 RNAi) (magnification × 100). (**B**). Isotype analysis showed cells were all consistently negative for CD34, Sca-1, and CD31 but positive for Flk1, CD29, CD44, and CD105. (**C**). AMSCs cultured in osteogenic, adipogenic or angiogenic media continuously, sustain their immunoregulatory activities. AMSCs (1 × 10^4^/well) were irradiated by 30 Gy after bone, adipose, and endothelial differentiation and then co-cultured with 50 μg/ml PHA stimulated lymphocytes (1 × 10^5^/well) for 2 days. 0.037 MBq/well ^3^H-TdR were added and cells incubated for 18 hours. Liquid scintillation was used to determined lymphocyte proliferation.

To characterize AMSCs, we first examined their morphology, differentiation ability, phenotype, and growth patterns. Isotype analysis indicated AMSCs were consistently negative for Sca-1, CD34 and CD31, but positive for Flk1, CD29, CD44, and CD105 (Fig. 1A). Results show that they consistently displayed fibroblast-like morphology and could differentiate into bone, fat, and cartilage, which indicated that the isolated cells had stem cell properties (Fig. 1B). AMSCs (1 × 10^4^/well) were irradiated by 30 Gy after bone, adipose, and endothelial differentiation and then co-cultured with 50 μg/ml PHA (polyhydroxyalkanoates) stimulated lymphocytes (1 × 10^5^/well) for 2 days. 0.037 MBq/well ^3^H-TdR were then added followed by incubation for 18 hours. Liquid scintillation was used to count lymphocyte proliferation. The results indicated that AMSCs cultured in osteogenic, adipogenic, or angiogenic media continuously sustained their immunoregulatory activities (Fig. 1C).

### AMSCs inhibit proliferation of PHA stimulated T lymphocytes

**Figure 2:**
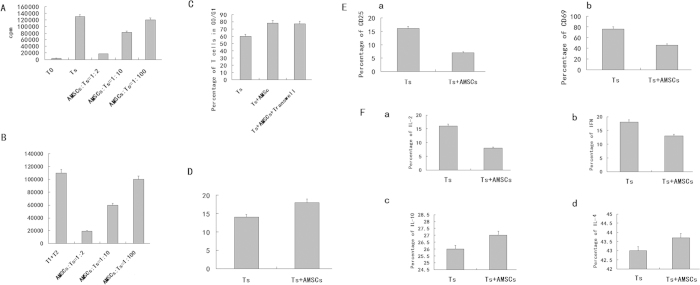
The effect of AMSCs on T lymphocyte proliferation. (**A**) The effects of AMSCs on T lymphocyte proliferation in mitogen proliferation assays. Three groups were assayed: non-stimulated T cells (none), PHA-stimulated T cells (Ts) and PHA-stimulated T cells co-cultured with MSC at different ratios (AMSC to T cell = 1:2, 1:10, 1:100). (**B**) The effect of AMSCs on T lymphocyte proliferation in MLR. AMSCs at a 1:10 ratio (irradiated MSCs to T cells). (**C**) Effects of AMSCs on T lymphocyte cell cycle. AMSCs or 3T3 at 1:10 ratios (MSCs to T cells). Cell cycle of PHA-stimulated T cells was analyzed for T cells alone (Ts), and co-cultured with AMSCs (aMSC + Ts). The 3T3 cell line was used as control (3T3 + Ts). Data are shown as mean±SD of five independent experiments. (**D**) Effect of AMSCs on T lymphocyte activation. AMSCs at 1:10 ratios (MSCs to T cells). T cell activation markers CD25, CD69, and CD44 were examined in T cells alone (Ts) and AMSCs co-cultured with activated T cells (AMSCs + Ts). **(E)** Effect of AMSCs on T cell apoptosis. AMSCs at a 1:10 ratio (AMSCs to T cells). (**F**) Effects of AMSCs on T cytokine secretion. The results showed that AMSCs could inhibit IL-2 and IFN-γ but had no significant effect on IL-10 and IL-4. These results indicate that under normal immune circumstances, AMSCs may inhibit Th0 to Th1 differentiation.

AMSCs inhibited T cells (lymphocytes) proliferation to below 1%, when co-cultured with T cells (T cell/AMSC ratio = 2:1). When T cells and MSCs were co-cultured at a ratio of 10:1, T cell inhibition was reduced, and T cell numbers were 63% the level of those with no MSCs or with PHA alone (*P* < 0.05). When AMSCs and T cells were co-cultured at a ratio of 100:1, the inhibitory effect disappeared completely (compared with PHA only, *P* > 0.05). These results suggested that AMSCs are able to inhibit PHA stimulated T cell proliferation in a dose dependent manner. Blocking of Jagged-2 in AMSCS attenuated the reduction of the T cell proliferation (Fig. 2A).

### AMSCs inhibit mixed lymphocyte reaction (MLR)

We collected peripheral blood mononuclear cells (PBMCs) from two healthy donors. Cells from one donor acted as stimulatory cells, while cells from the other were the effector cells. These cells were co-cultured with AMSCs in varying ratios. Significant inhibition was found when the ratio of mononuclear cells to AMSCs was of 2:1 (statistically significant compared to no co-culture, p < 0.05), with cell numbers reaching 16% of co-culture only levels in the case of AMSCs. When the ratio was 10:1, inhibition increased to 65% (compared to no co-culture, p < 0.05), and when the ratio was 100:1, the inhibition disappeared. These results suggest that AMSCs have an inhibitory effect on the MLR and that this effect is dose dependent and this effect is not affected by Jagged-2 interference (Fig. 2B).

### AMSCs inhibit T cell proliferation and the impact on Transwell

We co-cultured AMSCs with T cells (aMSC:T cell = 1:10) and examined proliferation 3 days later. The results show that AMSCs inhibit T cells in the G0/G1 phase, with the percent of cycling cells decreasing from 94.23 ± 2.26% to 61.27 ± 2.97% when co-cultured (Fig. 2C). There existed an obvious statistical difference compared with PHA stimulated T cells (p < 0.05). We next tested whether AMSCs inhibited T cell proliferation via secreted factors using a Transwell plate to separate AMSCs and T cells and record cell phase. The ratio of T cells in the G0/G1 phase was about 84% and there was no difference between with those with or without Transwell separation.

### Effects of AMSCs on T cell apoptosis

We used the Annexin V kit to examine T cell apoptosis when co-cultured with AMSCs. The percent of T cells undergoing apoptosis was 13.77 ± 0.68% in the absence of AMSCs but 10.07 ± 1.45% in the presence of AMSCs (*P* <  0.05) (Fig. 2D). These results suggest that AMSC inhibition of T cell proliferation was not due to T cell apoptosis. The Jagged-2 interference in AMSCs has no effects on the T cell apoptosis.

### Effects of AMSCs on early activation of T cells

To investigate AMSC inhibition of early T cell activation, we used flow cytometry to examine CD69 expression on T cells at 12 hours, and CD25 expression at 24 hours after PHA stimulation. AMSCs were able to inhibit CD69 (58.76 ± 4.83% to 11.06 ± 3.08%, p < 0.05). AMSCs could inhibit expression of CD25, decreasing expression from 13.5 ± 3.77% to 3.26 ± 1.76%, although this was not statistically significant, p > 0.05). The Jagged-2 interference in AMSCs has no effects on the T cell apoptosis. These results suggest that AMSCs inhibit early activation of T cells (Fig. 2E).

### Effects of AMSCs on T cytokine secretion

Induction of Th0 cells into Th1 cells is triggered mainly by IFN-γ and IL-2. Th0 induction into Th2 occurs through secretion of IL-10 and IL-4, stimulated by certain signals. To further elaborate the effects of AMSCs on Th0 cell differentiation into Th1 and Th2 cells, we co-cultured T lymphocytes with AMSCs and used ELISA to test the level of IL-10, IL-4, IL-2 and IFN-γ (Fig. 2F). AMSCs could inhibit IL-2 and IFN-γ but had no significant effect on IL-10 and IL-4. The same results were found after the Jagged-2 interference in AMSCs. These results indicate that under normal immune circumstances, AMSCs may inhibit Th0 to Th1 differentiation.

### The expression of MHC on chondrogenic differentiation or IFN-γ pretreated AMSCs

**Figure 3:**
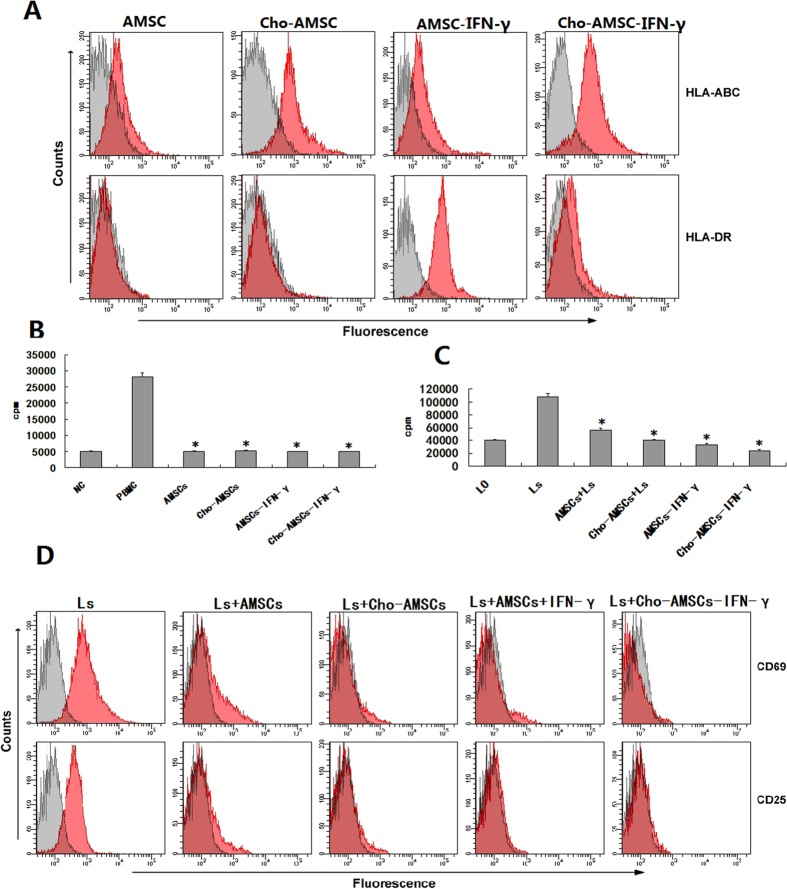
Immunological analysis of AMSCs after 7 days of chondrogenic differentiation or 48 hours of IFN-γ pretreatment. (**A**) FACS analysis for HLA expression in AMSCs following 7 days of chondrogenic differentiation or 48 hours of IFN-γ pretreatment. (**B**) AMSCs do not elicit a proliferative response from allogeneic lymphocytes after chondrogenic differentiation and/or IFN-γ pretreatment. (**C**) Inhibition of mitogen-stimulated lymphocyte proliferation by AMSCs increased after chondrogenic and/or IFN-γ pretreatment. (**D**) Suppression of mitogen-stimulated lymphocyte activity by AMSCs is retained after osteogenesis and/or IFN-γ pretreatment.

Flow cytometry analysis showed that AMSCs expressed low levels of MHC-I but did not express MHC-II. MHC-I expression was significantly upregulated after chondrogenic induction but MHC-II was still not expressed. AMSCs maintained low-level MHC-I expression after incubating with IFN-γ for 2 days, but MHC-II expression increased significantly (Fig. 3A).

### Enhanced effects of chondrogenic differentiation or IFN-γ pretreated AMSCs on the inhibition of lymphocyte proliferation

Previous studies have found that MSCs inhibit lymphocyte proliferation, and that this inhibition was in a MHC independent, but dose-dependent manner. Our results showed that the co-culture of AMSCs and lymphocytes reveal immunological characteristics similar to previous reports. Interestingly, although some reports showed IFN-γ stimulated MSCs could up-regulate MHC expression, we found here that AMSCs exhibited low-level immunogenicity but could not stimulate allogeneic lymphocyte proliferation after 7 days of chondrogenic differentiation or 48 hours of IFN-γ pretreatment (Fig. 3B). Inhibition of chondrogenic differentiation or IFN-γ pretreated AMSCs on PHA-stimulated lymphocyte proliferation was significantly enhanced in mitogen stimulation experiments (Fig. 3C). These results indicated that AMSCs can maintain low immunogenicity and enhanced ability to inhibit lymphocyte proliferation after chondrogenic differentiation or IFN-γ pretreatment.

### Enhanced effects of chondrogenic differentiation or IFN-γ pretreated AMSCs on the inhibition of lymphocyte activation

Co-culture of lymphocyte and AMSCs inhibited expression of the lymphocyte activation markers CD69 and CD25, and expression was significantly decreased by PHA stimulation but strengthened by chondrogenic differentiation or IFN-γ pretreatment. These results suggests that AMSCs inhibit lymphocyte activation (Fig. 3D).

### Effects of chondrogenic differentiation or IFN-γ pretreated AMSCs on IL-10 and TGF-β expression

**Figure 4:**
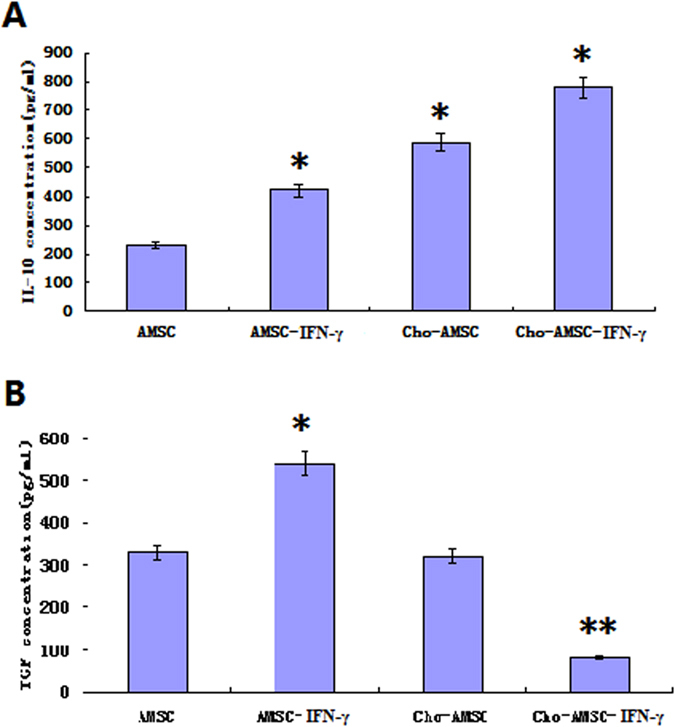
Chondrogenic differentiated AMSCs secrete more IL-10, but less TGF-β after IFN-γ pretreatment. (**A**) ELISA was used to detect IL-10 in supernatants from MSCs, Cho-MSCs, MSCs_IFN-gamma_, and Cho-MSCs_IFN-gamma_. (**B**) ELISA was used to detect TGF-β in supernatants from MSCs, Cho-MSCs, MSCs_IFN-gamma_, and Cho-MSCs_IFN-gamma_.

AMSCs secrete a variety of inhibitory cytokines that regulate lymphocyte immunity. We used ELISA to detect IL-10 and TGF-β in supernatants from MSCs, Cho-MSCs, MSCs_IFN-gamma_, and Cho-MSCs_IFN-gamma_. We found chondrogenic differentiated AMSCs secreted more IL-10 and that IFN-γ enhanced secretion. We did not detect any difference in TGF-β secretion between chondrogenic differentiated and undifferentiated AMSCs. Interestingly, IFN-γ stimulation upregulated TGF-β secretion from undifferentiated AMSCs but decreased TGF-β secretion from chondrogenic differentiated AMSCs (Fig. 4).

### Chondrogenic differentiation or IFN-γ stimulated AMSCs enhanced Jagged-2 expression

**Figure 5:**
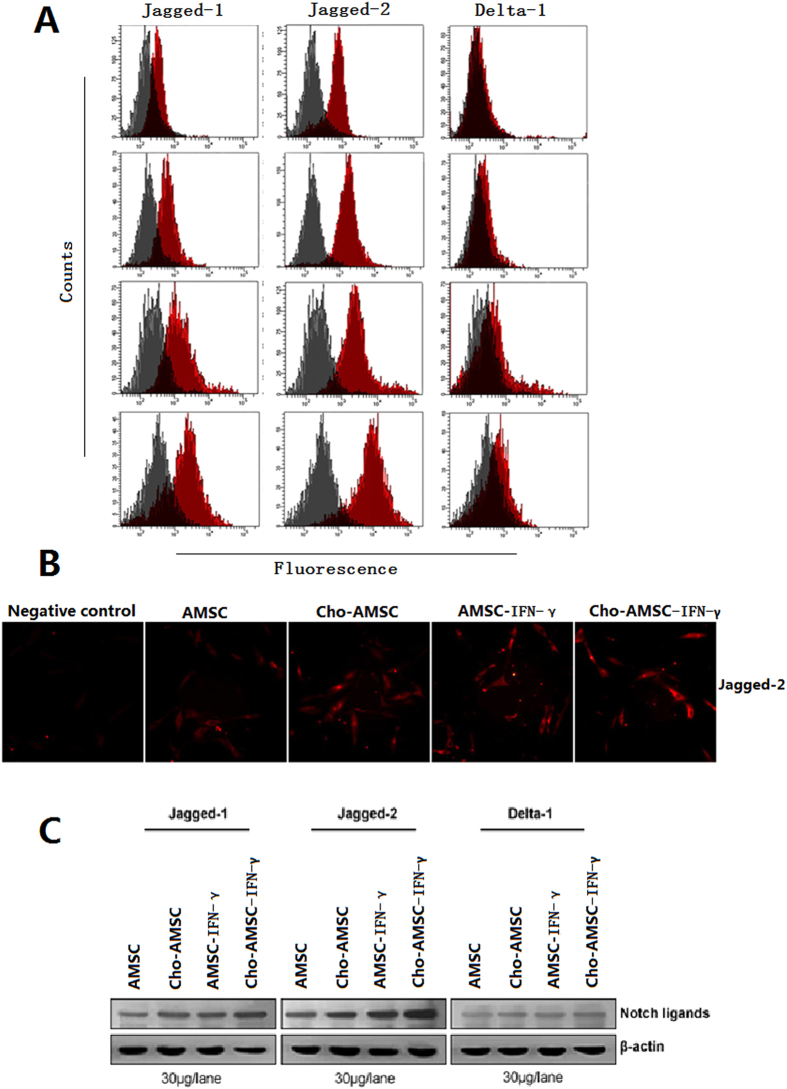
Chondrogenic differentiated and/or IFN-γ pretreated MSCs up-regulate Jagged-1. (**A**) FACS analysis of Notch ligand expression.(**B**) Indirect immunofluorescence staining detects Jagged-2. (**C**) Western blot analysis detects Notch ligand expression.

To determine whether the low immunogenicity and enhanced immunomodulatory activity of chondrogenic differentiation or IFN-γ stimulated AMSCs correlated with changes of Notch ligand, we examined ligand expression in each group of cells. We found Jagged-2 expression was significantly elevated following chondrogenic differentiation or IFN-γ pretreatment (Fig. 5A,B). Jagged-2 expression also increased, as determined by indirect immunofluorescence staining (Fig. 5C). This suggests that reduced immunogenicity and enhanced immunomodulatory activity from chondrogenic differentiation or IFN-γ pretreatment of AMSCs might be due to upregulation of Jagged-2.

### The immunological characteristics of chondrogenic differentiation or IFN-γ treated AMSCs depends on Jagged-2 to a certain degree

**Figure 6:**
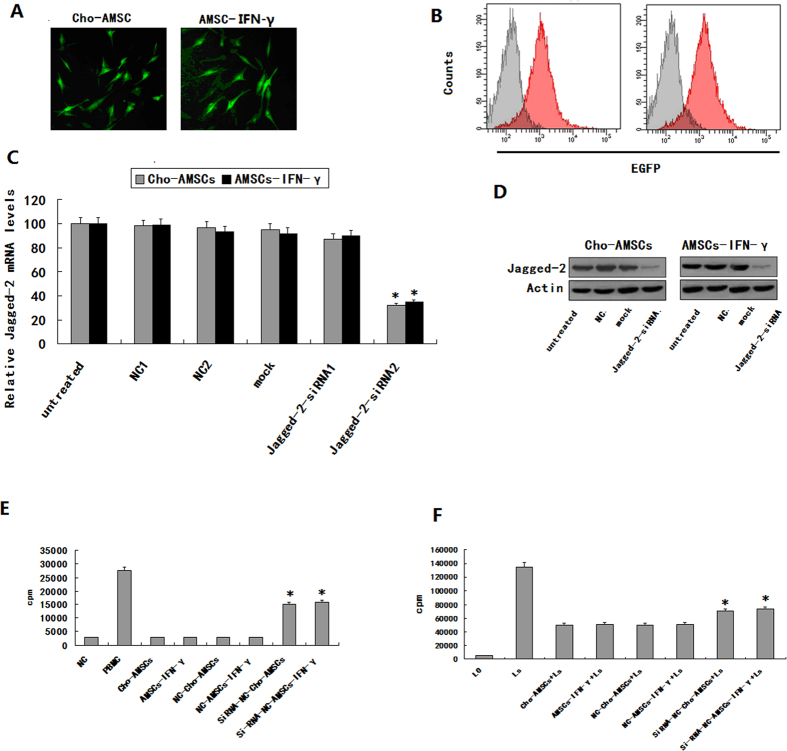
Immunologic characteristics of chondrogenic differentiated and/or IFN-γ pretreated AMSCs are dependent on up-regulation of Jagged-2. Jagged-2 mRNAi transfection efficiency of chondrogenic differentiated and/or IFN-γ pretreated AMSCs was measured using several methods (**A–D**). (**A**) Fluorescent microscopy. (**B**) FACS. (**C**) Real-time PCR. (**D**) Western blot. (**E**) The proliferation of allogeneic lymphocytes stimulated in MLC experiments of chondrogenic differentiated and/or IFN-γ pretreated AMSCs was enhanced after Jagged-2 mRNAi transfection (*P < 0.05). (**F**) Chondrogenic differentiated and/or IFN-γ pretreated AMSC proliferation was inhibited following PHA stimulation of lymphocytes (*P < 0.05).

We conducted Jagged-2-RNA interference experiments to test whether the immunological characteristics of chondrogenic differentiation or IFN-γ treated AMSCs might be associated with upregulation of Jagged-2. Results suggest that Jagged-2-siRNA2 suppresses Jagged-2 expression during chondrogenic differentiation and in IFN-γ pretreated AMSCs. Fluorescence microscopy (Fig. 6A) and flow cytometry (Fig. 6B) were used to determine transfection efficiency and real-time PCR (RT-PCR) (Fig. 6C) and western blot analysis (Fig. 6D) were used to evaluate interference efficiency. Mixed lymphocyte culture (MLC) and mitogen stimulation experiments further supported the notion that AMSC immunogenicity was enhanced by chondrogenic differentiation or IFN-γ treatment, and that Jagged-2 interference attenuated immunosuppressive activity (Fig. 6E,F). We therefore conclude that the immunological properties following chondrogenic differentiation or IFN-γ pretreated AMSCs depend, at least in part, on Jagged-2.

## Discussion

MSCs are widely studied as new therapeutic tools for a number of clinical applications^15^,^16^. Indeed, they are known to have differentiation capacities as well as paracrine effects via the secretion of growth factors, cytokines, and antifibrotic or angiogenic mediators. Adipose tissue is an abundant source of MSCs and has shown promise in the field of regenerative medicine^17^. Furthermore, MSCs are readily harvested in large numbers with low donor-site morbidity^18^. Over the past decade, numerous studies have generated preclinical data on the safety and efficacy of AMSCs, supporting potential use in future clinical applications^19^,^20^,^21^.

Several studies indicate that MSCs possess an immunosuppressive function both *in vitro* and *in vivo*^22^,^23^,^24^. However, the molecular mechanisms regulating the immunological properties of implanted MSCs in a differentiated microenvironment or surrounded by inflammatory factors are not fully understood. Despite reports showing that chondrogenic, osteogenic, and adipogenic differentiation of MSCs did not stimulate allogeneic lymphocyte proliferation even in the presence of upregulated MHC molecules^24^,^25^,^26^,^27^, they still had an immunomodulatory effect when treated with inflammatory cytokines such as IFN-γ. The molecular mechanisms regulating this process are not known. We used chondrogenic differentiation and a IFN-γ pretreatment model to mimic the *in vivo* induction and inflammatory microenvironment to observe changes in AMSC immunological characteristics and to examine the molecular mechanisms involved.

We found that chondrogenic differentiation or IFN-γ pretreated AMSCs maintained a reduced immunogenicity and an ability to inhibit lymphocyte proliferation and activation by secreting more IL-10. In addition, chondrogenic differentiation or IFN-γ pretreated AMSCs upregulated Jagged-2 molecule expression, and RNA interference experiments revealed AMSCs might maintain their immunological activity through a Jagged-2-dependent mechanism. Furthermore, AMSC immunosuppressive activity was enhanced after Jagged-2 upregulation and tissue differentiation and Jagged-2. We further inferred that terminal cells from specific lineages are likely to maintain their original immunomodulatory activity *in vivo* after differentiation of AMSCs, and that this maintenance is Jagged-2-dependent to some extent. This immunomodulatory role appears to protect AMSCs derived terminal cells from being eliminated by the host immune system.

Our data indicate that AMSCs maintain their original immunological characteristics to prevent a host-graft reaction, even if they are induced to differentiate into bone, cartilage, fat, and endothelial cells. The immunomodulatory activity of differentiated and undifferentiated AMSCs was enhanced by IFN-γ. Although AMSCs do not naturally express MHC-II, IFN-γ was able to upregulate MHC-II to make AMSCs a new type of antigen-presenting cell (APC). It had been reported that IFN-γ pre-treated AMSCs play the same APC role. Nevertheless, AMSCs did not lose their immunosuppressive properties following MHC-II upregulation. In contrast, immunomodulatory activity was enhanced. Jagged-2 RNA interference experiments clearly indicated that enhanced immunosuppressive activity correlated with upregulation of surface Jagged-2. We thereby propose that AMSCs enhance MHC-II and Jagged-1 expression during an inflammatory immune response. They stimulate T cells to express MHC antigen and interact with Jagged-2 on AMSCs and transform into Treg or Th2 to drive a humoral response. AMSCs maintain immunosuppressive activity by upregulating Jagged-2 during an inflammatory reaction.

AMSCs can differentiate into bone, cartilage, fat, and endothelial tissue in different microenvironments *in vitro*^28^. Generally, it takes 3 weeks to induce AMSCs into full-fledged terminal cells^29^. We detected the immunological properties of AMSCs 2 or 3 weeks after induction and results indicated that they did not change significantly with an extension of induction time, a continuous increase of surface Jagged-2 on AMSCs was observed. This appears to be an important mechanism for AMSCs to maintain long-term survival without rejection by the host immune system and sheds some light on how AMSCs might be used for transplantation without causing immune rejection.

We found that AMSCs inhibit T lymphocyte proliferation and that the effects on mitogen stimulation and T lymphocytes proliferation were dose dependent. Moreover, the inhibitory effect was significant when T lymphocytes and AMSCs were co-cultured at a ratio of 2:1 and disappeared when the ratio was 100:1. AMSCs inhibited more T lymphocytes in the G0/G1 phase but also inhibited early activation as well. AMSCs played a role in the differentiation of Th0 to Th1 or Th2, primarily by inhibiting differentiation of Th0 to Th1 cells (IL-2 and IFN-γ producing cells), but had little effect on differentiation of Th0 to Th2 cells (IL-4 and IL-10 producing cells), suggesting that their effects on the normal immune system is mainly through the cellular immune response.

Human adipose tissue is a promising alternative source of stem cells, and autologous AMSCs may lead to novel clinical applications. The immunoregulatory activity of AMSCs, which are dependent upon Jagged-2, might be stronger after allogeneic MSCs differentiate into the committed cells, suppressing rejection.

## Materials and Methods

### Reagents

AMSCs were grown in Dulbecco’s modified Eagle’s medium (L-DMEM), IMDM, DF12 (Life Technologies, San Diego, CA, USA), with platelet-derived growth factor BB (PDGF-BB, Sigma, St. Louis, MO, USA). The following antibodies were used: CD11a, CD29, CD31, CD34, CD44, CD45, CD73, CD105, CD106, CD166, CD184, HLA-ABC, HLA-DR (BD Biosciences, San Jose, CA, USA); anti-rabbit IgG- fluorescein isothiocyanate (FITC; Sigma), anti-mouse IgG-FITC (Sigma). Jagged-1 (Calbiochem, San Diego, CA); Jagged-2 (Chemicon, Temecula, CA); Delta-1(Santa Cruz Biotechnology, CA).

### Specimens

Adult bone marrow and adipose samples were taken from Beijing Shijitan Hospital, Capital Medical University (Beijing, China). Informed consent was obtained from all donors according to procedures approved by the Ethics Committee.

### Preparation of AMSCs cells from adult human fat

Human raw lipoaspirates from donors undergoing selective suction-assisted lipectomy were collected. The procedures were performed as described previously with some modifications^15^. The raw liposuctioned aspirate was washed extensively with D-Hanks solution to remove contaminating blood and local anesthetics. The cells were washed two times and plated in T-75 tissue culture flasks at a density of 2 × 10^6^/ml. Expansion medium contained 57% DMEM/F-12, 40% MCDB-201, 2% fetal calf serum, 10 ng/ml epidermal growth factor, 10 ng/ml platelet-derived growth factor BB, 100 U/ml penicillin, and 100 g/ml streptomycin. Once adherent cells were more than 70% confluent, they were detached with 0.125% trypsin and 0.01% EDTA, and replated at a 1:3 dilution under the same culture conditions.

### Fluorescence activated cell sorting (FACS)

For immunophenotype analysis, expanded clonal cells were stained with antibodies against Flk1, CD29, CD31, CD34, CD44, CD45, and CD106. For intracellular antigen detection, cells were first fixed in 2% paraformaldehyde for 15 minutes at 4°C and permeabilized with 0.1% saponin for 1 hour at room temperature. Cells were washed and labeled with fluorescein isothiocyanate (FITC) conjugated secondary goat anti-mouse, goat anti-rabbit, or sheep anti-goat antibodies (Sigma), then washed and analyzed using a FACSCalibur flow cytometer (BD, Franklin Lakes, NJ, USA).

### RNA-i experiments

The si-RNA sequence targeting human Jagged-2 (from mRNA sequence; Invitrogen online) corresponds to the coding region 377–403 relative to the first nucleotide of the start codon (target = 5′-AAC ATC ACC TAT TGG ATC CAA ACT AC-3′). Computer analysis using the software developed by Ambion Inc. confirmed this sequence to be a good target. si-RNAs were 21 nucleotides long with symmetric 2-nucleotide 3′overhangs composed of 2′-deoxythymidine to enhance nuclease resistance. The si-RNAs were synthesized chemically and high pressure liquid chromatography purified (Genset, Paris, France). Sense si-RNA sequence was 5′-CAUCACCUAUUGGAUCCAAdTdT-3′. Antisense si-RNA was 5′-UUGGAUCCAAUAGGUGAUGdTdT-3′. For annealing of si-RNAs, mixture of complementary single stranded RNAs (at equimolar concentration) was incubated in annealing buffer (20 mM Tris-HCl pH 7.5, 50 mM NaCl, and 10 mM MgCl_2_) for 2 minutes at 65 °C followed by a slow cooling to room temperature (at least 25 °C) and then proceeded to storage temperature of 4 °C. Before transfection, cells cultured at 50% confluence in 6-well plates (10 cm^2^) were washed two times with OPTIMEM 1 (Invitrogen) without FCS and incubated in 1.5 ml of this medium without FCS for 1 hour. Then, cells were transfected with Jagged-2 RNA duplex formulated into Mirus *Trans*IT-TKO transfection reagent (Mirus Corp, Interchim, France) according to the manufacturer’s instructions. Unless otherwise described, transfection used 20 nM RNA duplex in 0.5 ml of transfection medium OPTIMEM 1 without FCS per 5 × 10^5^ cells for 6 hours and then the medium volume was adjusted to 1.5 ml per well with RPMI 2% FCS. SilencerTM negative control 1 si-RNA (Ambion Inc.) was used as negative control under similar conditions(20 nM).The efficiency of silencing is 80% in our assay.

### Effect of AMSCs on T cell cycle

MSCs and MNCs (mononuclear cells) were prepared as described previously^11^. Briefly, T cells were stimulated with PHA (50 ug/ml, final concentration) for 3 days, alone or cocultured with MSCs (derived from normal and MDS patient) or 3T3 cells, then harvested and quantified. One million T cells were fixed with 70% cold ethanol at 4 °C for 30 min, washed twice with PBS, and stained with 50 ug/ml PI (Sigma) at room temperature for 5 minutes. Data were analyzed with Mod-FIT software (BD Biosciences).

### Effect of AMSCs on T cell activation

MSCs and MNCs were prepared as described above. T cells were cultured alone or co-cultured with prepared MSCs and stimulated with PHA (50 ug/ml final concentration). CD25 and CD69 expression was detected by flow cytometry at 24 h, and CD44 was detected at 72 h.

### Effect of AMSCs on T cell apoptosis

T cells were cultured alone or with MSCs stimulated by PHA (50 ug/ml final concentration) for 3 days, then harvested and quantified, stained with Annexin-V kit (BD, USA), and analyzed by flow cytometry (FACS Vantage, BD Biosciences).

### Enzyme-linked Immunoadsorbent Assays (ELISA)

ELISA assays were performed according to the manufacturer’s recommendations (Oncogene Research Products, EMD Biosciences, Billerica, MA, USA). Results were compared with those obtained from serially diluted solutions of commercially purified controls. Anti-human cytokine antibodies were added at 0.4 ug/ml in 0.05 M bicarbonate buffer (pH 9.3) to 96-well, U-bottom, polyvinyl microplates, and 1 × 10^5^ cells/100 μl. After incubation for 1 hour at 37 °C, plates were washed, and 50 ng/ml biotinylated anti-mouse antibody was added for 1 hour at 37 °C. The plates were washed and incubated with streptavidin-HRP for 1 hour at 37 °C. After washing, 0.2 mM ABTS was added to the wells, incubated for 10 minutes, and the colorimetric reaction measured at 405 nm by ELISA (VERSAmax).

### Mitogen proliferative assays

In mitogen proliferation assays, triplicate wells containing 1 × 10^5^ responder blood mononuclear cells (MNCs) were cultured with 50 ug/ml PHA (Roche, Indianapolis, IN, USA) in 0.1 ml medium at 37 °C in 5% CO_2_, and Flk1^+^CD31^−^CD34^−^ AMSCs were added on day 0. Irradiated Flk1^+^CD31^−^CD34^−^ AMSCs (30Gy) were co-cultured with the MNCs at different ratios (MSCs to MNCs = 1:2, 1:10, 1:100). Control wells contained only MNCs.

### Mixed lymphocyte reaction assays (MLR)

MNCs were prepared from normal donor peripheral blood by Ficoll-Paque density gradient centrifugation and suspended in RPMI 1640 medium supplemented with 10% (vol/vol) FCS, 2 mM L-glutamine, 0.1 mM nonessential amino acids, 1 mM sodium pyruvate, and 100 U/mL penicillin.

### Western blot and Immunoprecipitation

AMSCs were harvested at specific times after treatment, as indicated. Cell lysates were mixed with loading buffer, electrophoresed, and proteins transferred to polyvinyl difluoride membranes (Filtron, Pall Corporation, Port Washington, NY, USA) using a semidry blotting apparatus (GE Healthcare BioSciences, Piscataway, NJ, USA) and probed with mouse mAbs, followed by incubation with peroxidase-labeled secondary antibodies. Detection was performed using a chemiluminescence system (GE Healthcare Biosciences) according to the manufacturer’s instructions. Membranes were striped with elution buffer and reprobed with antibodies against the non-phosphorylated protein as a loading control. Immunoprecipitation controls used the same procedure, except agarose beads contained only mouse IgG.

### Ethical Statement

All the methods were carried out in accordance with the approved guidelines approved by Shantou University, Medical College Ethnical Care Committee. All experimental protocols were approved by a Guangdong Medical College Ethnical Care Committee.

### Statistical analysis

Results are expressed as mean ± SD. Data were analyzed using the unpaired two-tailed student’s t test and the log rank test. *P* < 0.05 was considered significant.

## Additional Information

**How to cite this article**: Xishan, Z. *et al.* Jagged-2 enhances immunomodulatory activity in adipose derived mesenchymal stem cells. *Sci. Rep.* **5**, 14284; doi: 10.1038/srep14284 (2015).
